# Plant size differences between quartz islands and their surroundings in a South African desert ecosystem

**DOI:** 10.1093/aobpla/plag047

**Published:** 2026-09-29

**Authors:** Severin D H Irl, Pia M Eibes, Alexander M Bürger, Katharina Meyer, Mareike Thier, Ute Schmiedel, Gianluigi Ottaviani

**Affiliations:** Biogeography and Biodiversity Lab, Institute of Physical Geography, Goethe-University Frankfurt, Altenhöferallee 1, 60438 Frankfurt am Main, Germany; Center of Interdisciplinary African Studies (ZIAF), Goethe-University Frankfurt, Norbert-Wollheim-Platz 1, 60323 Frankfurt am Main, Germany; Senckenberg Biodiversity and Climate Research Centre (BIK-F), Senckenberganlage 25, 60325 Frankfurt am Main, Germany; Biogeography and Biodiversity Lab, Institute of Physical Geography, Goethe-University Frankfurt, Altenhöferallee 1, 60438 Frankfurt am Main, Germany; Center of Interdisciplinary African Studies (ZIAF), Goethe-University Frankfurt, Norbert-Wollheim-Platz 1, 60323 Frankfurt am Main, Germany; Biogeography and Biodiversity Lab, Institute of Physical Geography, Goethe-University Frankfurt, Altenhöferallee 1, 60438 Frankfurt am Main, Germany; Center of Interdisciplinary African Studies (ZIAF), Goethe-University Frankfurt, Norbert-Wollheim-Platz 1, 60323 Frankfurt am Main, Germany; Institute of Plant Science and Microbiology, University of Hamburg, Ohnhorststraße 18, 22609 Hamburg, Germany; Biogeography and Biodiversity Lab, Institute of Physical Geography, Goethe-University Frankfurt, Altenhöferallee 1, 60438 Frankfurt am Main, Germany; Institute of Plant Science and Microbiology, University of Hamburg, Ohnhorststraße 18, 22609 Hamburg, Germany; School for Climate Studies (SCS), Stellenbosch University, Natural Sciences Building, Merriman Avenue, 7600 Stellenbosch, South Africa; Centro de Investigaciones sobre Desertificación, Consejo Superior de Investigaciones Científicas, Universitat de València, Generalitat Valenciana (CIDE, CSIC-UV-GV), Carretera CV-315 km. 10.7, 46113 Valencia, Spain

**Keywords:** edaphic islands, endemism, functional island biogeography, habitat specialization, leaf size, plant height, Succulent Karoo biome, trait co-variation

## Abstract

Islands constitute excellent models to disentangle the effect of different biotic and abiotic factors generating plant size variation. However, plant size variation in terrestrial habitat islands remains unexplored. This is a meaningful gap as these insular systems are expected to have both similarities (e.g. isolation defined by habitat contrast and permeability against the surrounding matrix) as well as differences (e.g. large herbivores’ presence). Here, we address this gap by examining size-related plant trait differences between specialist species of terrestrial habitat islands (quartz islands) and species not restricted to the islands. We collected size-related traits (plant height, leaf length, area and volume) for 67 species belonging to the three most representative families (Aizoaceae, Asteraceae, Crassulaceae) in the Richtersveld desert, South Africa. Species were categorized as quartz specialists (restricted to the islands), generalists (occurring on islands and matrix) and matrix species (found only in the matrix). We ran univariate, bivariate and multivariate analysis to examine whether quartz specialists differ in trait values and coordination compared to generalists and matrix species. Quartz specialists are consistently more variable in their plant size traits, are shorter, yet are characterized by similar leaf size (and succulence) compared to generalists and matrix species. The variation of whole-plant stature is decoupled from that of leaf traits. Our findings suggest that environmental filtering associated with quartz islands’ harsh conditions (low resource availability and solar radiation) and related ecological specialization may operate as the main eco-evolutionary driver shaping size-related traits, selecting for compact (dwarf) succulent growth forms.

## Teaser

Terrestrial habitat islands can strongly shape plant size and growth form. In the harsh quartz islands of South Africa’s Richtersveld, specialist species are consistently shorter and show greater variation in plant size than generalist and matrix species, while maintaining similar leaf size and succulence. This decoupling of whole-plant and leaf traits suggests that environmental filtering favours compact, dwarf succulent growth forms as an ecological specialization.

## Introduction

The study of scaling relationships between biomass or traits of different organs—examined through co-variation analysis—informs on what can generate variation in plant size, constituting a key research focus in plant ecology and evolution (Niklas 1995, West et al. 1999, Moles et al. 2005, 2009). This research line can disentangle whether, for example, the variation of a size-related trait (e.g. plant height) correlates with variation in stem, leaf, seed or root, such that those changes may or may not carry an evolutionary meaning (adaptive or not; e.g. Burns et al. 2012, Biddick et al. 2019a, Smith 2020). Biotic and abiotic factors related to main eco-evolutionary processes can then contribute to shaping trait co-variation. These factors involve: (i) biomechanical constraints (e.g. Niklas 1993, 1995, West et al. 1999), (ii) ontogeny (Laurans et al. 2024, Millan et al. 2024), (iii) intra- and interspecific responses (e.g. Midolo et al. 2019, 2024, Fajardo et al. 2020, Ottaviani et al. 2025), (iv) disturbance regimes (e.g. fire, herbivory; Moncrieff et al. 2011, Millan et al. 2024), (v) climate or soil conditions (e.g. Midolo et al. 2019, Westerband et al. 2023), and (vi) biogeographic context (e.g. latitude or island vs mainland systems; Burns et al. 2012, Burns 2016, Biddick et al. 2019b).

Islands represent suitable model systems to examine evolutionary size changes in organisms—as in the case of the island rule (Foster 1964, Van-Valen 1973). This research interest and effort was initially directed towards animals (Foster 1964, Lomolino 1985, Meiri et al. 2006) then extended to plants (Carlquist 1974, Biddick et al. 2019b, Biddick and Burns 2021, Moreira et al. 2026). Studies from South Pacific oceanic islands showed how plants tend to align with those predictions for plant stature and leaf size but not for seed size (Kavanagh and Burns 2014, Cox and Burns 2017, Biddick et al. 2019b, Biddick and Burns 2021). Mixed results emerged for flower size depending on the pollination syndrome (Ciarle et al. 2025), with patterns also differing between growth forms (woodies vs herbs; Biddick and Burns 2021). Plant size changes observed on island species can be therefore associated with multiple eco-evolutionary processes linked to insularity, such as (i) reduced interspecific competition and intensified intraspecific competition (Whitman and Aarssen 2010, Burns et al. 2012, Burns 2019), (ii) limited presence or even lack of disturbance associated with large herbivores (Burns 2016, 2019), (iii) greater water storage capacity and therefore a more efficient water-use on resource-poor islands (Biddick and Burns 2021).

The concept of insular systems extends to other systems than oceanic islands, namely terrestrial habitat islands, such as inselbergs or edaphic islands (Harrison et al. 2006, Itescu 2019, Ottaviani et al. 2020, Méndez-Castro et al. 2021, Vanschoenwinkel et al. 2025). Like oceanic islands, terrestrial habitat islands are spatially isolated entities characterized by limited area, spatial and temporal isolation and low connectivity between units embedded in a dissimilar landscape matrix (Itescu 2019, Méndez-Castro et al. 2021). Such a biogeographic setting (resembling that of oceanic islands and archipelagos) may affect plant size patterns, yet this topic remains underexplored on terrestrial habitat islands (but see Ottaviani et al. 2019, Conti et al. 2022), especially if considered in a scaling relationship framework.

At the same time, terrestrial habitat islands differ fundamentally from oceanic islands in several putative ecological, evolutionary and biogeographical attributes (Ricketts 2001, Ottaviani et al. 2020). The degree of spatio-temporal isolation experienced by organisms on terrestrial habitat islands depends primarily on the landscape permeability, defined as the environmental contrast between the habitat island and the surrounding landscape matrix (Stamps et al. 1987, Méndez-Castro et al. 2021, Eibes et al. 2025). Landscape permeability can largely affect species’ movement, colonization likelihood and gene flow between populations (Schmidt et al. 2025). This is because dispersal ability varies between species as well as between populations or even individuals within a population, potentially increasing the relevance of processes such as the rescue effect (Brose 2003). As a result, species’ habitat specialization is a key feature because the conceptual framework offered by functional island biogeography (*sensu* Ottaviani et al. 2020, Schrader et al. 2021a) would only apply to those taxa that are specialized and restricted to the patch of focal habitat type serving as putative island, namely that are expected to be affected by insularity to some degree (e.g. Conti et al. 2022, Ottaviani et al. 2022, Midolo et al. 2024). Another difference between oceanic and terrestrial habitat islands refers to disturbance types and regimes. Differences in landscape permeability implies that disturbance agents that are absent or rarely present on oceanic islands are instead similarly occurring on terrestrial habitat islands and the surrounding matrix, such as exposure to large herbivores (Burns et al. 2012, Burns 2019). Finally, climate buffering provided by water in oceanic islands (Burns 2019) is expected to play no role for terrestrial habitat islands, which are instead often characterized by markedly harsh environmental conditions, such as high solar irradiance, toxicity and poor-resource soils typical of edaphic islands (Harrison et al. 2006, Damschen et al. 2012, Méndez-Castro et al. 2021, Eibes et al. 2022, Ottaviani et al. 2022). Soil chemical or physical properties defining edaphic islands are indeed in stark contrast with those prevailing in the surrounding matrix.

Here, we set out to examine this underexplored topic by analysing plant size differences of species specialized to a model type of terrestrial habitat islands (edaphic islands), namely quartz islands found in the Richtersveld region (northwestern South Africa). This is a highly diverse mountainous desert area supporting a high degree of endemism (van Wyk et al. 2024) within the Succulent Karoo biome (Mucina et al. 2014). The so-called edaphism (or edaphic endemism; Mota et al. 2017, Eibes et al. 2022) results in a large number of habitat specialists having unique life forms such as dwarf succulents (Boucher et al. 2017, Eibes et al. 2021, 2022). We collected data on size-related traits (plant height, leaf size) for 67 species belonging to the three most representative families in the region (Aizoaceae, Asteraceae, Crassulaceae), dealing with interspecific differences. We compared whether plant stature (height) and leaf size differ between groups of habitat specialization, namely quartz island specialists (occurring only on the quartz edaphic islands), generalists (occurring both on the islands and the landscape matrix) and matrix species (found only in the surrounding Succulent Karoo). As proxies for leaf size, we included three traits: leaf length, area, volume—the latter also functionally indicative of leaf succulence and water storage capacity, which is a particularly important strategy in the desert study region and biome. Specifically, we ask:

(**Q1**) Does plant size differ between species belonging to different groups of habitat specialization?
(**Q2**) Do scaling relationships occur between plant height and leaf size, and do these relationships differ across species belonging to different families or habitat specialization groups?

## Materials and methods

### Study area

The Richtersveld is part of the species-rich Namaqualand-Namib Domain of the Lowland Succulent Karoo Biome, which is dominated by leaf-succulent shrubs (Jürgens 1991). Among the 25 global biodiversity hotspots, the Succulent Karoo stands out as the only one located entirely in an arid region and is therefore considered the world's most diverse desert (Myers et al. 2000). It harbours 0.6% of the world's known plant species and is predicted to host a high number of undiscovered endemic species (Myers et al. 2000). Globally, the Succulent Karoo desert is the only arid region with winter rainfall dominated by leaf-succulent shrubs (Mucina et al. 2014). The northern part of the Richtersveld is defined by a rugged mountainous topography, which progressively transitions into moderately undulating to flat terrain in the south (Schmiedel 2002). In the Richterveld, intensive weathering of quartz veins has led to the development of fields covered in quartz gravel, the quartz islands (Schmiedel and Jürgens 1999).

The Richtersveld shows an arid desert climate, classified as a BWh-Köppen-Geiger climate (Beck et al. 2018). The cold Benguela Current along the Atlantic Coast causes descending air masses, which forms a stable inversion layer that inhibits vertical air movement and suppresses cloud formation and precipitation in the Richtersveld (Nußbaum 2003). The region lies in the winter rainfall zone (Schmiedel 2002), with a mean annual temperature of ∼18.2°C and mean annual precipitation of ∼108 mm, with the most precipitation falling between June and August (climate-data.org). In addition to the rainfall, the cold ocean surface promotes the formation of fog, which is transported inland by the Atlantic-Benguela anticyclone, especially during winter, providing an extra source of humidity to the system (Nußbaum 2003).

The quartz islands in the Succulent Karoo exhibit harsh edaphic conditions such as low water-holding capacity, low nutrient content and shallow soils in comparison to the surrounding environments (Schmiedel et al. 2015). As a result, these quartz islands have a unique plant species composition that differs from the surrounding Succulent Karoo matrix (Eibes et al. 2021). Many plant species are highly specialized and endemic to the quartz island habitat (Schmiedel and Jürgens 1999, Eibes et al. 2022). The colonization of the quartz islands within this specialist species group likely took place multiple times independently (Schmiedel 2002, Zhigila et al. 2024). In the Succulent Karoo, Aizoaceae, Asteraceae and Crassulaceae are particularly rich in species on the quartz islands compared to the matrix vegetation (Eibes et al. 2022). The Succulent Karoo displays a wide diversity of growth forms, with many highly branched chamaephytes and nano-phanerophytes (Schmiedel 2002). In contrast, the growth forms on the quartz islands differ significantly from the surrounding matrix because they harbour compact growth forms close to the surface such as nano-chamaephytes smaller than 5 cm (Schmiedel 2002, Eibes et al. 2022).

### Sampling design

A survey was conducted on 37 quartz islands as well as the surrounding landscape matrix (Fig. 1). On these quartz islands and in the matrix, we then recorded size-related plant traits (see next section). The quartz islands were initially pre-identified through a supervised satellite image classification (*sensu* Oldeland et al. 2022) and field validated. Species occurrences were collected using a walk-through method, which involves systematically crossing each quartz island and the surrounding matrix for extended periods of time until no new species appeared (Johnson et al. 2008, Vetaas et al. 2014). The time spent per island averaged to about 2h per hectare. This method has been successfully applied to quartz islands in different regions of southern Africa that have led to new insights into the island biogeography of this system (Eibes et al. 2025, Bürger et al. 2026). In this study quartz islands are defined as surfaces with at least 50% quartz gravel coverage (Eibes et al. 2025). Field sampling took place in August and September 2024. We deposited herbarium specimens in Bolus Herbarium (BOL) in Cape Town, the Herbarium Hamburgense (HBG) and the Natural History Museum of Vienna (W).

**Figure 1:**
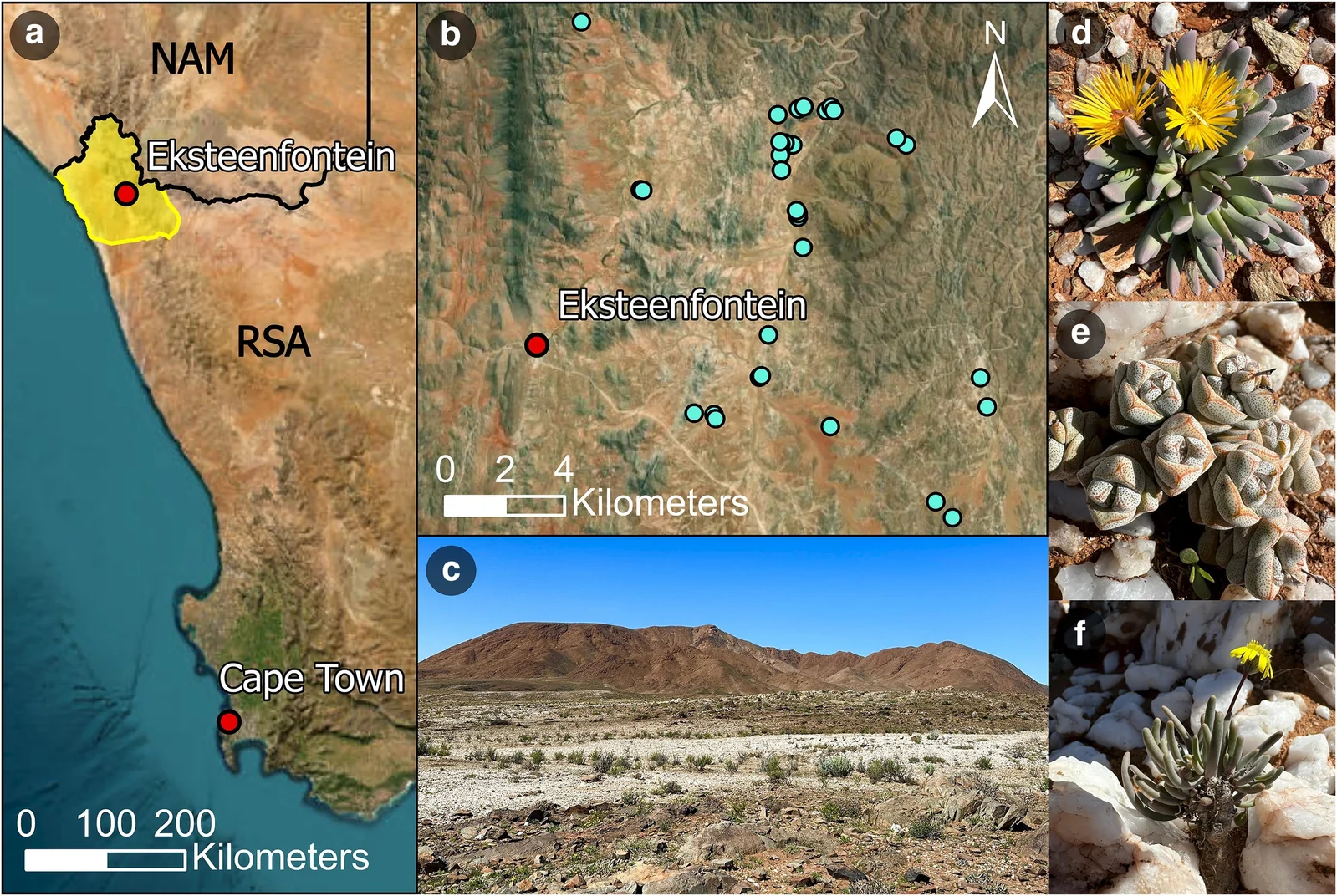
Study area and examples of species belonging to most representative quartz island families. a) The location of the Richtersveld (yellow) in the Northern Cape of South Africa, b) the 37 sampled study sites (turquoise points) near the town of Eksteenfontein, c) quartz islands in the mountainous study area in the Richtersveld, d) *Cheiridopsis robusta* (Aizoaceae), e) *Crassula deceptor* (Crassulaceae), f) *Crassothonna protecta* (Asteraceae). (Photos: Mareike Thier; Basemap Sources: Esri, Earthstar Geographics).

### Trait measurement

Trait data were collected from the three most abundant and representative families found on the quartz islands—Aizoaceae, Crassulaceae, Asteraceae—for 67 perennial species. Altogether we found 44 Aizoceae, 12 Crassulaceae and 11 Asteraceae. Based on extensive field observations, expert knowledge and literature, each species was categorized based on its habitat requirement (specialization), namely as quartz specialist (occurring only on quartz islands), generalist (occurring on both quartz islands and in the surrounding landscape matrix), and matrix species (occurring only in the landscape surrounding the quartz islands). We classified 37 species as quartz specialists, 22 as generalists and eight as matrix species (a link to the complete species list including family, habitat specialization and the measured trait values is given in the data publication statement). Because of conservation concerns (including highly threatened endemic species), we attempted to limit the sampling impacts on local populations. Therefore, we collected trait data from three individuals per species, hence focusing on interspecific trait differences. To reduce potential bias from site-specific microenvironmental conditions, each individual of a species was sampled from a different quartz island or matrix site.

Plant height was measured perpendicularly from the ground to the highest point, excluding inflorescences (Bürger et al. 2026). For leaf traits (length, area, volume) we collected between two to 30 leaves per individual, depending on plant morphology and leaf size. In some nano-chamaephyte species *sensu* Schmiedel and Jürgens (1999), such as *Conophytum maughanii subsp. latum*, entire individuals consisted of only one leaf pair, which could be sampled. In most cases we sampled five leaves. However, for species with particularly small leaves (e.g. *Eriocephalus microphyllus*), we collected up to 30 leaves to ensure sufficient biomass for further analysis (leaf scans). We measured leaf length using a digital calipre. Two-dimensional leaves were scanned with petioles to determine the leaf area using the software ImageJ (Schneider et al. 2012). For three-dimensional leaves, we measured leaf length and width using a digital calipre. Then, we approximated their shape to basic geometric shapes such as ellipsoids, prisms and (semi-)cylinders to calculate their leaf area and leaf volume based on the geometric equation for each shape. We provide an overview of the trait values for the full dataset of the three most important families and for Aizoaceae only (Table 1). In addition, we calculated the ratio between log10(leaf length) and log10(plant height) as well as between log10(leaf volume) and log10(plant height), representing direct indicators of leaf size (length) and succulence (volume) variation in relation to plant stature, respectively.

**Table 1:** Minimum, maximum and mean (raw) values of the four size-related traits (plant height, leaf length, leaf area, leaf volume, the ratio between leaf length and plant height) considered in this study for the full dataset (including all three families) and for the Aizoaceae species only.

	*Full dataset* (*n* = 67)			*Aizoaceae only* (*n* = 44)		
	Min	Max	Mean ± sd	Min	Max	Mean ± sd
Plant height [mm]	6.9	1001.7	238.9 ± 179.6	6.9	736.7	224.5 ± 160.3
Leaf length [mm]	1.9	97.7	24.9 ± 19.0	3.1	62.8	23.5 ± 15.4
Leaf area [mm^2^]	1.1	18 413.6	1589.8 ± 3094.2	9.4	7444.4	909.9 ± 1396.8
Leaf volume [mm^3^]	0.3	159 779.3	6909.2 ± 24 673.2	5.7	8051.4	1514.2 ± 2265.1
Leaf length / Plant height	0.01	2.11	0.21 ± 0.32	0.02	2.11	0.23 ± 0.36

### Data analysis

We ran all the analyses in R, version 4.5.3 (R Core Team 2026). Because all matrix species in our dataset belong to one family (Aizoaceae; accounting for ∼65% of all species), we ran all the analyses described below to tackle our two research questions both for the entire dataset and for Aizoaceae species only—this way qualitatively checking whether such phylogenetic unbalance in the sampling had an effect on the observed trait patterns for the three habitat specialization (quartz specialists, generalists, matrix species). We performed a Principal Component Analysis (PCA; scaled data) on the four log10-transformed size-related traits (plant height, leaf length, leaf area, leaf volume) as a preliminary step to explore trait co-variation (using functions available in the *vegan* package; Oksanen et al. 2020).

For comparing size-related trait patterns across habitat specialization groups we performed ANOVA test comparing mean and variability for each size-related trait (including log10(leaf length)/log10(plant height) and log10(leaf volume)/log10(plant height) ratios) among the three habitat specialization groups and applying Tukey HSD post hoc test to evaluate pairwise differences between groups, if model outputs conferred with variance homogeneity. We ran these tests using the package *stats* (R Core Team, 2026). We used Levene-Tests to test for homogeneity of variance for each trait between groups of habitat requirement using the *car* package (Fox and Weisberg 2019).

To explore whether scaling relationships between plant height and leaf size occur, and if these differ among different families and habitat specialization groups—we ran standardized major axis (SMA) linear regression using functions available in the *smatr* package (Warton et al. 2012). SMA is the most suitable regression method when examining bivariate scaling links, because it does not assume that X predicts Y, affecting the slope and intercept estimate (Warton et al. 2006, 2012). We set either families or habitat specialization as grouping factors in the SMA models to extract group-specific summary statistics (slope and its 95% confidence intervals, R^2^, *P*-value) of the scaling.

## Results

### Comparing size-related trait patterns across habitat specialization groups

The multivariate analysis (PCA) ran on the size-related traits for the full dataset illustrates how the three leaf traits (length, area, volume) are correlated to some degree and ordered along the first axis (explaining 71% of model variability for the full dataset; 69% for Aizoaceae species only; Fig. 2). Plant height instead is mainly related to the second axis (explaining 24% of model variability for the full dataset; 24% for Aizoaceae species only), so forming an orthogonal functional independent dimension (Fig. 2). Crassulaceae species tend occupy a larger size-related space than the other two families (Fig. 2a). Quartz specialists and matrix-derived species tend to occupy a distinct functional space compared to matrix species, with generalists sitting in-between these two groups (in both the full dataset and Aizoaceae only; Fig. 2b and c)—with quartz specialists having a larger occupancy.

**Figure 2:**
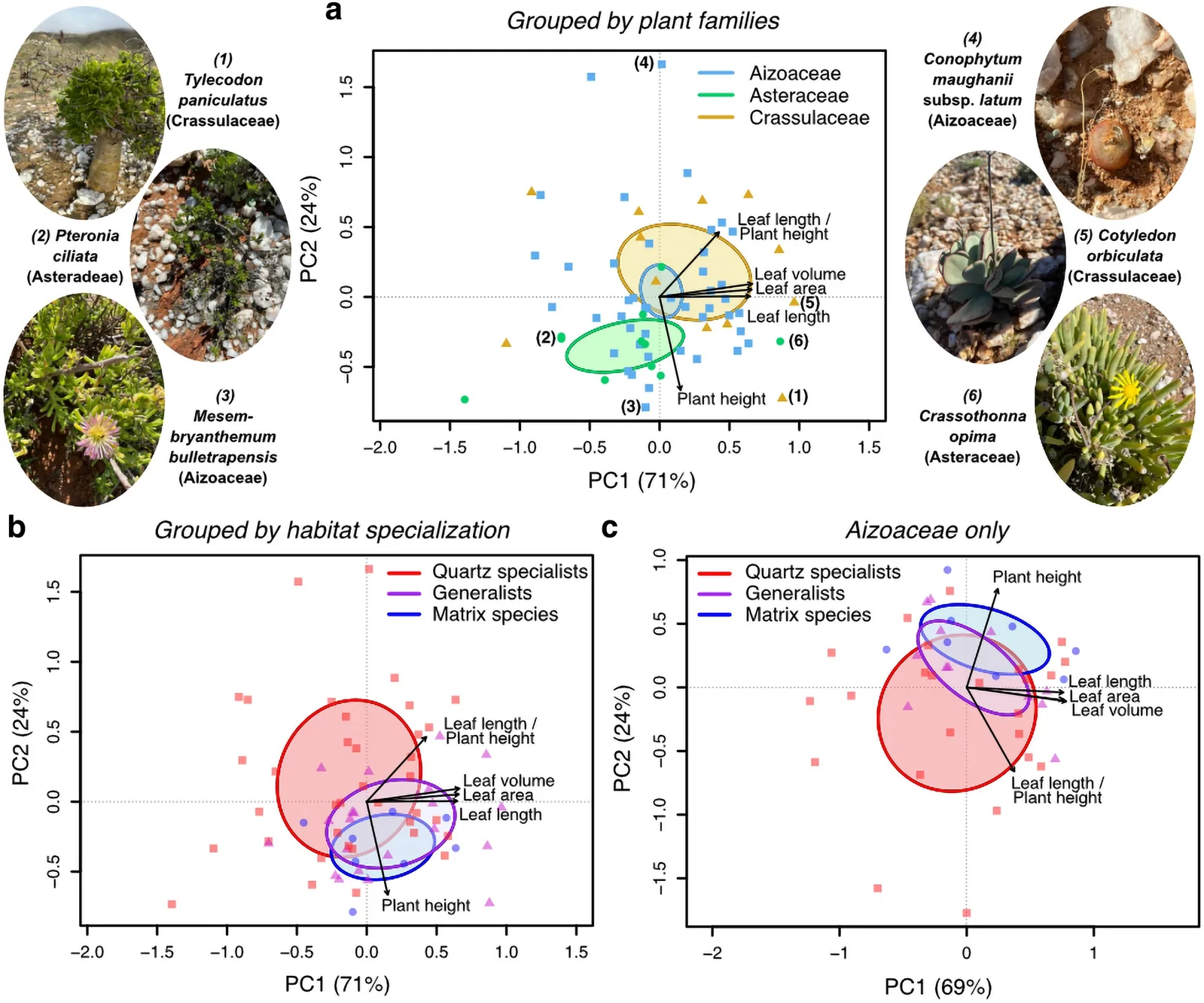
PCA grouped by a) families and b) habitat specialization based on full dataset (*n* = 67 species). c) PCA for the most important family (Aizoaceae)—the only family that has species in all three habitat specialization groups (*n* = 44 species). In a) six representative species in the Richtersveld belonging to the three main families highlight their characteristics occupancy by species in the size-related trait space of quartz islands and their surroundings. All variables in the PCA are log10-transformed. Pictures © Mareike Thier.

The univariate analysis comparing plant size-related traits across the three groups of habitat specialization indicates, however, that, for the full dataset, quartz specialists tend to be shorter than generalists and matrix species (Fig. 3a), whereas generalists tend to have longer leaves than quartz specialists (Fig. 3b). The ratio between leaf length and plant height does not vary among groups (Fig. 3c). For the Aizoaceae-only dataset, quartz specialists tend to be shorter than matrix species, with generalists having intermediate statures (Fig. 3d). Leaf size of Aizoaceae species belonging to different habitat specialization groups instead does not differ significantly (Fig. 3e; Supplementary Figure S1, Supplementary Table S2). Leaf length to plant height ratio (Fig. 3f) also does not vary across habitat specialization groups. Summary statistics for all these models are reported in Supplementary Table S3. The homogeneity of variance analysis indicates that quartz island specialists are characterized by consistently higher variation of size-related traits compared to generalists and matrix-derived species, especially for plant height—with the pattern emerging both for the full dataset and for Aizoaceae only (Table 2).

**Figure 3:**
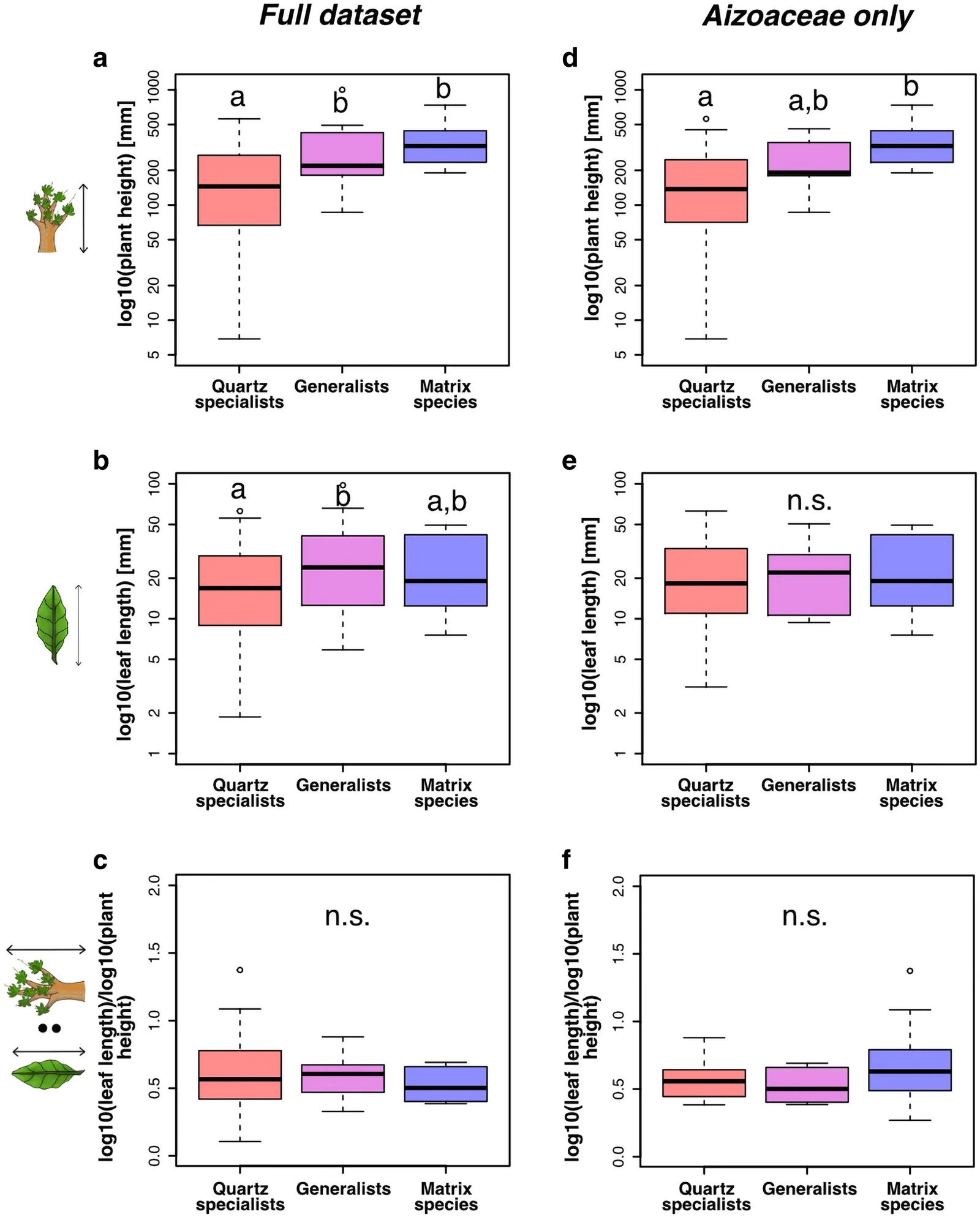
Comparison of plant height, leaf length and the ratio of leaf length to plant height by groups of habitat specialization for the full dataset (a–c) and Aizoaceae only (d–f). Lower case letters indicate significant differences between groups based on ANOVA (Supplementary Table S3). Trait icons © Pia Eibes.

**Table 2:** Levene-Test of homogeneity of variance for the full dataset as well as for Aizoaceae only for plant height, leaf length and the ratio between leaf length and plant height.

		*Full dataset (n* = *67)*			*Aizoaceae only (n* = *44)*		
	Degrees of freedom	log10 (plant height)	log10(leaf length)	log10(leaf length) / log10(plant height)	log10 (plant height)	log10(leaf length)	log10(leaf length) / log10(plant height)
Levene-Test	2	**P** **=** 0.02*	P = 0.57 n.s.	**P** **=** **0.09.**	**P** **=** **0.05.**	P = 0.38 n.s.	P = 0.22 n.s.
Quartz specialists vs. generalists	1	**P** **=** 0.03*	0.41	**P** **=** **0.08.**	**P** **=** **0.06.**	0.19	0.20
Quartz specialists vs. matrix species	1	**P** **=** **0.07.**	0.45	P = 0.15 n.s.	**P** **=** **0.09.**	0.55	0.20
Matrix species vs. generalists	1	P = 0.45 n.s.	0.78	P = 0.52 n.s.	P = 0.88 n.s.	0.56	0.70

The Levene-Test was first run to test for differences in the homogeneity of variance for all three habitat requirement groups. Each habitat requirement group was compared to each other in a separate Levene-Test. Alpha was set at 0.1 (*P*-values: * ≤ 0.05; . ≤ 0.1; n.s. = not significant > 0.05). Significant differences are highlighted as bold text in the table.

### Examining scaling relationships between plant height and leaf size across families and habitat specialization groups

The SMA analysis testing for size-related scaling relationships between plant height and leaf length shows that variation of these two plant traits is independent (Fig. 4; Supplementary Table S4). This complete decoupling between plant height and the two size-related leaf traits occurs either when the full dataset is considered or grouped by families (Fig. 4a) and habitat specialization (Fig. 4b) or when dealing with Aizoaceae only (Fig. 4c)—a lack of relationship confirmed also for leaf area and leaf volume (Supplementary Figure S5; Supplementary Table S6).

**Figure 4:**
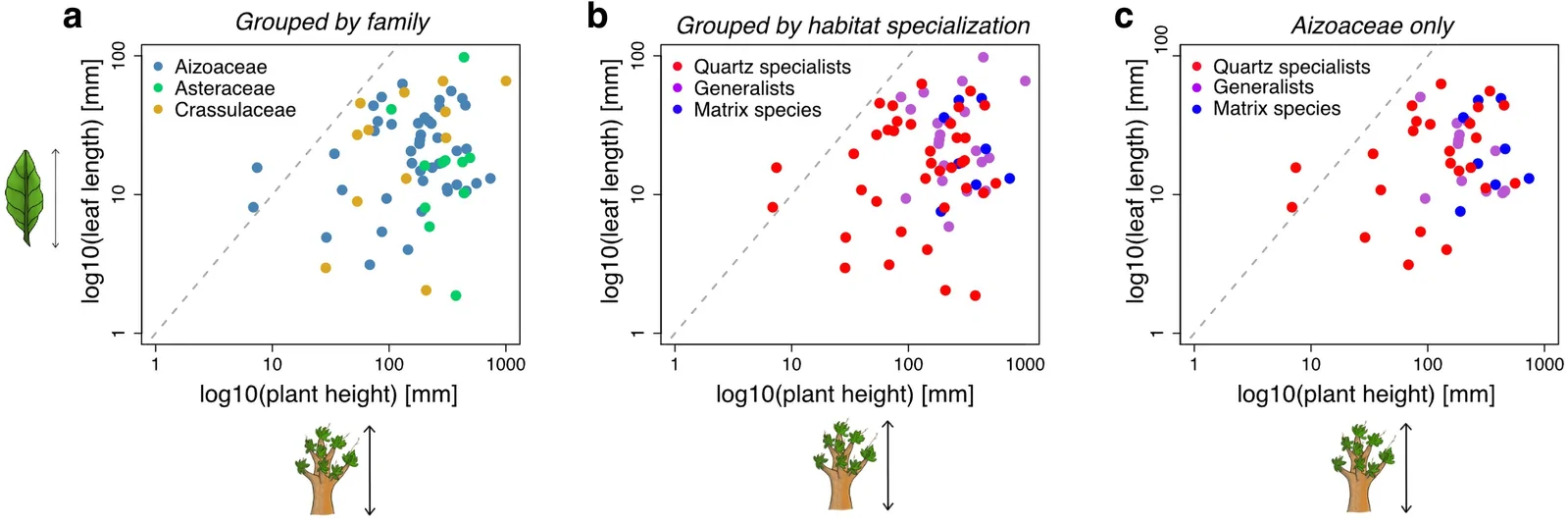
Size-related scaling relationship (lack thereof) between leaf length and plant height. a) Species are grouped by plant families, b) by habitat specialization and c) by habitat specialization for Aizoaceae only. The dashed line indicates isometry, namely slope = 1. Trait icons © Pia Eibes.

## Discussion

Our findings indicate that plant species specialized and restricted to the Richtersveld desert quartz islands tend to be consistently shorter but with similar leaf sizes than species occurring both on quartz and the landscape matrix (generalists) or found only in the surrounding Succulent Karoo habitats (matrix species) (Figs. 2, 3, 4; Table 2). This pattern may be linked to a putative main eco-evolutionary filter acting on specialist species that are fine-tuned to cope with harsh environmental conditions associated with shallow and resource-poor soils and high solar irradiance that define quartz habitat islands (Schmiedel and Jürgens 1999, Schmiedel et al. 2015). This strong environmental filter may favour plants characterized by more compact (close-to-surface) dwarf and succulent forms—as observed also in quartz island specialist species from other regions experiencing similar eco-evolutionary conditions (e.g. Eibes et al. 2021, 2022, Bürger et al. 2026, Meyer et al. 2026). Our results also stress the importance of preserving quartz islands’ habitat adaptations associated with functional uniqueness—paralleled by evolutionary and floristics distinctiveness of the study region and biome (Mucina et al. 2014, Eibes et al. 2022).

### Quartz specialists are shorter but have similar leaf size than generalists and matrix species

The main pattern that consistently emerges across the comparisons between habitat specialization groups of size-related traits refers to shorter plants on the quartz islands, but this difference does not emerge for leaf size (Fig. 3; Table 2). A reduced stature together with a comparable leaf size (length, area, volume) of quartz specialists vs generalists or matrix species may indicate that the eco-evolutionary pressure (environmental filter) associated with continuously high solar irradiance and low soil resource availability (water, nutrients) defining quartz islands may select for growth forms and functional strategies that reduce evapotranspiration by forming compact dwarf succulent forms close to the soil surface (Eibes et al. 2021, 2022). The evidence of compact forms and resource-conservative functional strategies associated with specialist species of quartz islands align with results emerging from other edaphic island systems worldwide—such as granite outcrop (e.g. Ottaviani and Keppel 2018, Ottaviani et al. 2022) or serpentine outcrops (e.g. Kazakou et al. 2008, Hidalgo-Triana et al. 2023)—that are also distinguished by harsh soil conditions compared to the surrounding landscapes.

Unexpectedly, we found that plant size traits tend to vary more among quartz-specialist species than they do among matrix or generalist species (Fig. 3; Table 2), suggesting that the strong edaphic filter defining the quartz islands may promote, rather than constrain, the diversity of functional strategies related to biomass allocation or water-use and storage (von Willert 1992). High microhabitat heterogeneity of the quartz islands (Schmiedel et al. 2015, Eibes et al. 2021)—associated with fine-scale edaphic and microclimate conditions (e.g. Palacio et al. 2025)—may play an important role in favouring high variation in plant size strategies (hence variable types of compact, dwarf and succulent forms) of quartz specialists.

### Plant height and size-related leaf traits are decoupled, with evolutionary history and environmental filtering playing a putative key role

Size-related leaf traits are coordinated in the multivariate functional space but are decoupled from plant height, forming two independent size-related functional dimensions (Figs. 2, 4). This evidence contrasts with allometric predictions that are often focused on woody, mostly tree species (Niklas 1993, 1995, Sun et al. 2006), i.e. larger plants having larger leaves. Succulent herbs or dwarf shrubs found in arid or hyper-arid desert ecosystems, as those included in this study, may, however, deviate from this expected pattern because they face different biomechanical constraints and different putative main eco-evolutionary forces (Boucher et al. 2017).

Species belonging to the three most representative families in the Richtersveld desert region are characterized by distinct space occupancy in the multivariate functional space identified by size-related traits (especially Asteraceae vs Crassulaceae and Aizoaceae; Fig. 2a). This indicates that species belonging to these three families tend to have distinct size-related niches. Additionally, species belonging to the three habitat specialization groups (quartz specialists, matrix species, generalists) differ among them in their occupancy of the multivariate functional space (Fig. 2b). This finding suggests that both evolutionary history (as also inferred from the phylogenetic structure of quartz island specialist assemblages; Zhigila et al. 2024) and ecological factors of the quartz habitat may have left a functional signature on the multivariate size-related trait coordination of the focal plant species—an inference further supported when examining Aizoaceae species alone (accounting for all matrix species in our dataset; Fig. 2c).

The bivariate analysis devised to examine whether scaling relationships occur between plant height and leaf size further confirms the complete functional decoupling—both across families and habitat specialization groups (Fig. 4). Even though no significant links are revealed between plant height and leaf length, most species tend to lump off the isometric line (i.e. slope = 1). This is challenging to interpret and contextualize in the study system, because it may indicate that the focal plant species have disproportionately smaller leaves for a given height (yet decoupled) or, the other way around, that they are characterized by a ‘giant’ stature for a given leaf size potentially associated with a loss of allometry (see Stillwell et al. 2016, Biddick et al. 2019b). At the same time, when examining bivariate plots between plant height and either leaf volume or area (both confirming the complete plant size-related trait decoupling), species are homogeneously distributed above and below the isometric line—suggesting a possible effect related to dimensionality and units, which may have affected the observed patterns. Leaf shape of succulent species (accounting for most of our species pool and defining the Succulent Karoo biome; Mucina et al. 2014) can contribute to this discrepancy in size-related leaf traits, namely between the one-dimensional leaf length vs two-dimensional leaf area vs three-dimensional leaf volume. Leaf volume better captures leaf succulence in the studied eco-evolutionarily context, yet in our dataset all these leaf size-related traits are collinear (Fig. 2). Still, by visually inspecting species’ location in the plots, quartz specialists are characterized by consistently shorter statures (i.e. all the shortest species are quartz specialists; indicating dwarfism) than generalists and matrix species (which instead include all larger species in the study system), corroborating previous findings (Eibes et al. 2022).

### Study limitations

Our study is not devoid of limitations. We recommend future studies to include a more balanced number of species across habitat specialization groups and families. We indeed acknowledge that our sampling design was unbalanced across habitat specialization groups and families, namely quartz island specialists are ∼5-fold more numerous than matrix species (all belonging to the Aizoaceae family in our dataset), and that Aizoaceae species comprise ∼65% of our studied species. At the same time, by examining size-related trait patterns for the full dataset as well as for Aizoaceae species only, we qualitatively accounted for this sampling bias, so we consider our findings and related inferences robust. Additionally, measurement accuracy of leaf length is inherently more accurate than leaf volume (calculated through multiple variables, including leaf length), so we encourage technical and methodological developments to bridge this accuracy gap—as similarly done for other leaf traits (e.g. Schrader et al. 2021b). We also suggest enlarging the list of plant traits, including other key functional strategies enabling plants to cope with harsh soil conditions typically defining edaphic islands, and tightly related to size (e.g. xeromorphy, leaf water content, rooting depth). This extension could also consider the role of intraspecific trait variability, which is increasingly recognized as fundamental in shaping potential plant adaptations and functional responses to environmental changes (e.g. Albert et al. 2011, Violle et al. 2012, Palacio et al. 2025).

### Concluding remarks and future directions

Our findings, taken together, suggest that the putative insularity of quartz islands plays a minor role in shaping size-related trait patterns of the focal plant species compared to the ecological role of environmental filtering associated with harsh desert conditions and species evolutionary history. Two possible reasons could explain this inference. First, large herbivores are present both in the landscape matrix and on the quartz islands—a key difference with oceanic islands wherein plants are typically released from this disturbance type (as those herbivores are less capable of long-distance dispersal compared to e.g. birds; Burns et al. 2012, Burns 2019). Herbivore trampling—more than herbivore eating-behavior, considering that many plant species in the study area are characterized by chemical defenses making them toxic or low palatable (Farmer and Milton 2006)—may contribute to selecting for compact dwarf forms. Also, plant species in the Succulent Karoo biome tend not to have zoochory as the main dispersal syndrome (Eibes et al. 2022), and only a few of those species (endozoochorous species) can germinate from dung seed banks. Second, long-term effects of continuous resource limitation may constitute a main challenge and selective force for plants; the hyper-diversity of succulent plant species and forms in the study area (as well as in the broader region and embedding biome; Mucina et al. 2014) further hints in that direction.

Future studies could compare size-related plant trait patterns from other quartz island systems, ideally extending to other types of terrestrial habitat islands (e.g. edaphic islands associated with granite, serpentine or gypsum outcrops) from other regions and biomes, searching for generalities. This replicated setting may also include oceanic islands, so testing for expected similarities and differences in plant size variation between oceanic islands and terrestrial habitat islands (e.g. Ottaviani et al. 2020). Additionally, examining plant size variation along insularity gradients may refine our view of insularity-trait links (Ottaviani et al. 2020, 2022, Midolo et al. 2024). These conceptual and methodological advances would move the field of plant functional biogeography of insular systems, especially of terrestrial habitat islands, considerably forward.

## Supplementary Material

plag047_Supplementary_Data

## Data Availability

All data (Supplementary Table S7) and code (Supplementary File S8) are available as an online supplement to this paper. However, the exact GPS coordinates of the sampled locations will remain undisclosed to avoid plant poaching.
