# Supplementary material for: Plant size differences between quartz islands and their surroundings in a South African desert ecosystem
Source: AoB Plants. 2026 Sep 29;18(5):plag047. doi: 10.1093/aobpla/plag047 (PMC13650701; doi:10.1093/aobpla/plag047)
Supplement: Supplementary file 1 [file plag047_supplementary_data.zip › Supporting Information.pdf]

Supporting Information

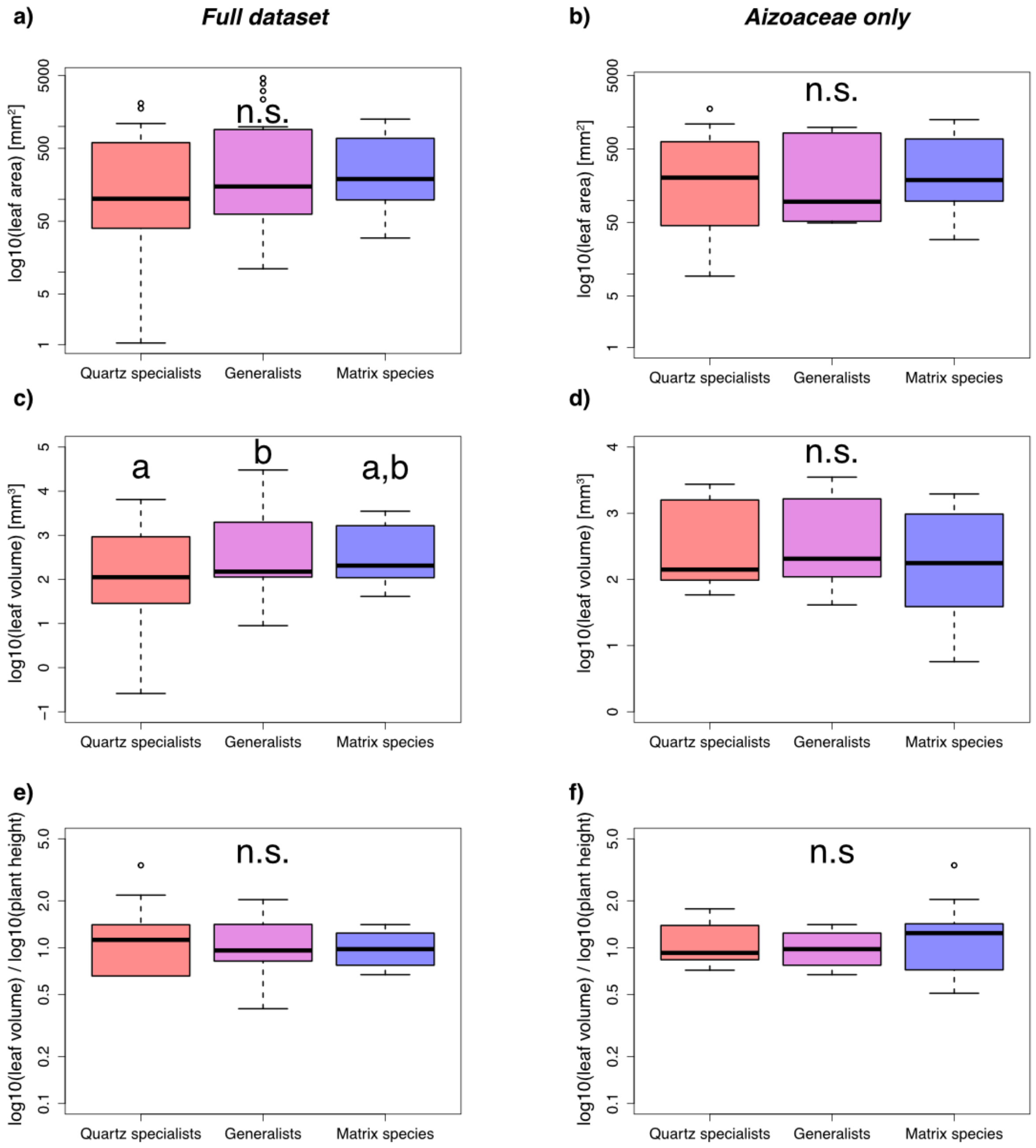

Figure S1. Comparison of leaf area, leaf volume and the ratio of leaf volume to plant height by groups of specialization for the full dataset (a) and Aizoaceae only (b). All relationships are non-significant (see Table S2 for details).

Table S2. Summary statistics for Fig. S1 for full dataset with all three families and for Aizoaceae only for leaf area, leaf volume and the ratio of leaf volume and plant height. Alpha was set at 0.1 owing to the rather small sample sizes.

	**Full dataset (n = 67)**		**Aizoaceae only (n = 44)**	
	log(leaf area)		log(leaf area)	
Full model	p = 0.19 n.s.	F = 1.69, sum of squares = 1.97	p = 0.87 n.s.	F = 0.12, sum of squares = 0.14
Quartz specialists vs. generalists	p = 0.19 n.s.		p = 0.98 n.s.	
Quartz specialists vs. matrix species	p = 0.62 n.s.		p = 0.86 n.s.	
Generalists vs. matrix species	p = 0.96 n.s.		p = 0.95 n.s.	

	**Full dataset (n = 67)**		**Aizoaceae only (n = 44)**	
	log(leaf volume)		log(leaf volume)	
Full model	**p = 0.09.**	F = 2.46, Sum of squares = 4.52	p = 0.61 n.s.	F = 0.50, Sum of squares = 0.57
Quartz specialists vs. generalists	**p = 0.09.**		p = 0.74 n.s.	
Quartz specialists vs. matrix species	p = 0.49 n.s.		p = 0.67 n.s.	
Generalists vs. matrix species	p = 0.95 n.s.		p = 0.98 n.s.	

	**Full dataset (n = 67)**		**Aizoaceae only (n = 44)**	
	log10(leaf volume)/log10(plant height)		log10(leaf volume)/log10(plant height)	
Full model	p = 0.88 n.s.	F = 0.12, Sum of squares = 0.20	p = 0.58 n.s.	F = 0.56, Sum of squares = 0.30
Quartz specialists vs. generalists	p = 0.99 n.s.		p = 0.76 n.s.	
Quartz specialists vs. matrix species	p = 0.90 n.s.		p = 0.61 n.s.	
Generalists vs. matrix species	p = 0.88 n.s.		p = 0.95 n.s.	

Table S3. Summary statistics related to trait patterns shown in Fig. 3 for full dataset with all three families and for Aizoaceae only for plant height, leaf length, leaf volume (all three log-transformed), the ratio between leaf length and plant height as well as between leaf volume and plant height. Text in bold refers to significant and marginally significant (at alpha ≤ 0.1, given the small sample size). Note that F-values result from parametric ANOVA (with Tukey HSD post-hoc) to compare habitat specialization groups. All trait values were log10-transformed prior to analysis.

Full dataset (n = 67)						
	log10(plant height)		log10(leaf length)		log10(leaf length)/log10(plant height)	
Full model	**p = 0.001**	F = 7.58 Sum of squares = 2.23	**p = 0.09**	F = 2.41 Sum of squares = 0.66	p = 0.69	F = 0.38, Sum of squares = 0.036
Quartz specialists vs. generalists	**p = 0.007**		**p = 0.09**		p = 0.97	
Quartz specialists vs. matrix species	**p = 0.009**		p = 0.56		p = 0.66	
Generalists vs. matrix species	p = 0.70		p = 0.91		p = 0.78	

Aizoaceae only (n = 44)						
	log10(Plant height)		log10(Leaf length)		log10(Leaf length)/log10(plant height)	

Full model	**p = 0.008**	F = 5.47, Sum of squares = 1.75	p = 0.84	F = 0.17 Sum of squares = 0.04	p = 0.26	F = 0.123 Sum of squares = 0.06
Quartz specialists vs. generalists	**p = 0.09**		p = 0.94		p = 0.45	
Quartz specialists vs. matrix species	**p = 0.011**		p = 0.84		p = 0.32	
Generalists vs. matrix species	p = 0.55		p = 0.97		p = 0.93	

19

Table S4. Summary statistics reporting model estimate (with 95% confidence intervals), strength (based on R^2) and significance level (p-value) of the relationship shown in Fig. 4 - extracted from standardized major axis regression between plant height and leaf length.

		Plant height vs Leaf length		
Family	***n***	***Estimate (95% CIs)***	R^2	***p***
Aizoaceae	44	0.73 (0.54, 0.99)	0.05	0.14
Asteraceae	11	-2.17 (-4.34, -1.08)	<0.01	0.84
Crassulaceae	12	1.14 (0.62, 2.08)	0.18	0.17
Habitat specialization	***n***	***Estimate (95% CIs)***	R^2	***p***
Quartz specialists	37	0.87 (0.63, 1.22)	0.02	0.44
Generalists	22	1.23 (0.79, 1.94)	<0.01	0.92
Matrix species	8	-1.51 (-3.65, -0.63)	<0.01	0.95
Habitat specialization (Aizoaceae only)	***n***	***Estimate (95% CIs)***	R^2	***p***
Quartz specialists	24	0.74 (0.49, 1.11)	0.12	0.12
Generalists	12	-1.06 (-1.91, -0.59)	0.21	0.13
Matrix species	8	-1.51 (-3.64, -0.63)	<0.01	0.95

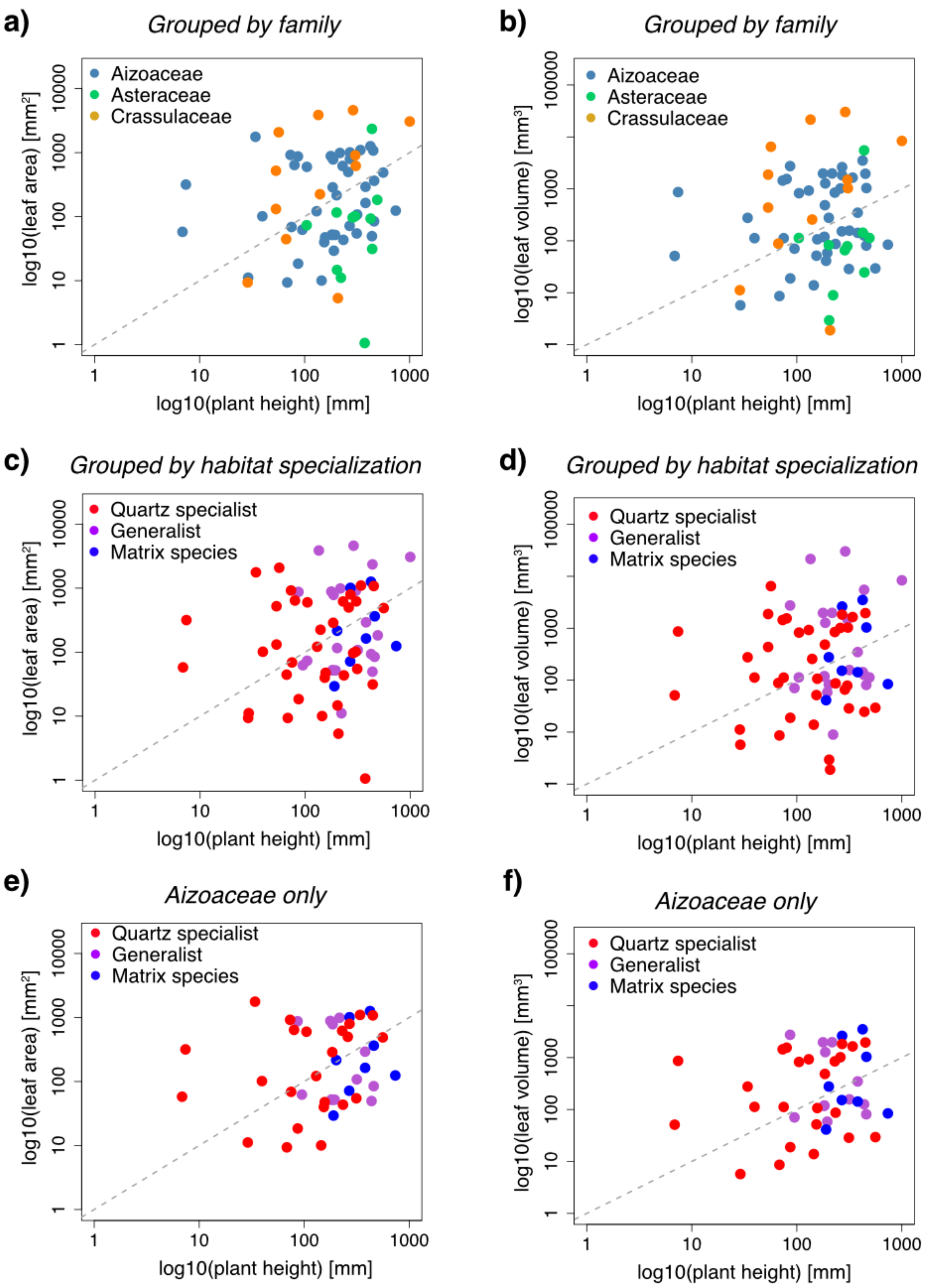

Figure S5. Size-related scaling relationship of leaf area vs. plant height in a), c) and e) and leaf volume vs. plant height in b), d) and f). Species are grouped by plant families (a-b), by habitat specialization (c-d) and by habitat specialization for Aizoaceae only (e-f). The dashed line indicates isometry, namely slope = 1. All relationships are non-significant (see details in Tab. S6).

Table S6. Summary statistics for plant height vs. leaf area and plant height vs. leaf volume - model estimate, 95% confidence intervals, strength of the relationship (based on R^2), significance level (p-value) - extracted from standardized major axis regression between plant height and leaf area (as reported in Fig. S5). All relationships are non-significant.

	Plant height vs Leaf area			***Plant height vs Leaf volume***		
**Family**	**Estimate (95% CIs)**	**R^2**	**p-value**	**Estimate (95% CIs)**	**R^2**	**p**
Aizoaceae	1.46 (1.08, 1.97)	0.03	0.26	1.70 (1.26, 2.31)	<0.03	0.29
Asteraceae	4.12 (2.07, 8.19)	0.03	0.63	5.38 (2.70, 10.74)	0.02	0.69
Crassulaceae	2.24 (1.24, 4.05)	0.21	0.13	2.90 (1.56, 5.40)	0.12	0.26
**Habitat requirement**	**Estimate (95% CIs)**	**R^2**	**p-value**	**Estimate (95% CIs)**	**R^2**	**p**
Quartz specialists	1.74 (1.25, 2.44)	<0.01	0.93	-2.19 (-3.06-, -1.56)	<0.01	0.65
Generalists	2.84 (1.82, 4.43)	0.02	0.50	3.57 (2.28, 5.59)	<0.01	0.69
Matrix species	2.78 (1.19, 6.52)	0.08	0.50	3.57 (1.49, 8.56)	0.02	0.74
**Habitat requirement (Aizoaceae only)**	**Estimate (95% CIs)**	**R^2**	**p-value**	**Estimate (95% CIs)**	**R^2**	**P**
Quartz specialists	1.45 (0.95, 2.20)	0.05	0.27	1.66 (1.09, 2.53)	0.03	0.40
Generalists	-2.42 (-4.57, -1.28)	0.07	0.42	-2.72 (-5.10, -1.45)	0.10	0.34
Matrix species	2.78 (1.19, 6.52)	0.08	0.50	3.57 (1.49, 8.56)	0.02	0.72
